# Bone Mineral Density in Schizophrenia: An Update of Current Meta-Analysis and Literature Review Under Guideline of PRISMA

**DOI:** 10.1097/MD.0000000000001967

**Published:** 2015-10-30

**Authors:** Ping-Tao Tseng, Yen-Wen Chen, Pin-Yang Yeh, Kun-Yu Tu, Yu-Shian Cheng, Ching-Kuan Wu

**Affiliations:** From the Department of Psychiatry, Tsyr-Huey Mental Hospital, Kaohsiung Jen-Ai's Home (PT, T, K-YT, Y-SC, C-KW), Department of Neurology, E-Da Hospital, Kaohsiung (YW, C), Institute of Allied Health Sciences, College of Medicine, National Cheng Kung University, Tainan (P-YY); and Department of Clinical Psychology, Tsyr-Huey Mental Hospital, Kaohsiung, Taiwan (P-YY).

## Abstract

Numerous reports have discussed bone mineral density (BMD) or the risk of osteoporosis in schizophrenia, but have yielded only controversial results.

We conducted an update of meta-analysis to examine the overall change in BMD in patients with schizophrenia and the effect on BMD of different antipsychotic drugs.

Electronic research through platform of PubMed.

The inclusion criteria were as follows: articles with relevance to comparisons of BMD in patients with schizophrenia (SCHIZ) and healthy controls (HCs), or articles discussing comparisons of BMD in SCHIZ receiving prolactin-raising (PR) and prolactin-sparing (PS) antipsychotics; articles about clinical trials.

In the current meta-analysis, we used the random-effect model to pool the results from 13 studies comparing BMD in SCHIZ and in HCs, and the results from 7 studies comparing BMD in patients receiving PR and PS.

Our results revealed significantly lower BMD in SCHIZ than in HCs (*P* < 0.001). In the meta-regression, mean age of subjects modulated the difference in BMD between patients and control subjects (*P* < 0.001). In addition, the BMD in SCHIZ taking PR was significantly lower than in those taking PS (*P* = 0.006).

Our study can only point to the phenomenon that BMD in SCHIZ is lower than that in HCs, and cannot reveal any possible pathophysiology or mechanism of this phenomenon. In addition, we could not rule out the possible effect of medication on BMD based on the results of the meta-analysis of comparison of BMD in SCHIZ receiving PR and PS.

The main result of our meta-analysis suggests that BMD is significantly lower in SCHIZ than in HCs. Our study emphasizes the importance of further screening for the risk of osteoporosis in young-aged schizophrenic patients, especially those taking PR, which are in high risk of fracture.

## INTRODUCTION

Osteoporosis is a major public health problem worldwide. It is characterized as gradually decreased bone mineral density (BMD) in systemic skeletons. People with osteoporosis are vulnerable to bone fracture, which can lead to disability and mortality. Many indices have been used with osteoporosis, and BMD is one of the most frequently applied. Also, several indicators have been used to describe BMD. Although the absolute raw levels are the most direct description of BMD, they are irrelevant in clinical settings because of the dynamic changes of levels associated with age, sex, and other clinical variables. The most relevant description of BMD is that using the *t* score or *z* score. The former indicates how many standard deviations one's BMD is above or below the mean BMD in a reference of “young-adult population”; the latter is a comparison of BMD with mean BMD in an “age- and sex-matched population.”^1^ Some techniques are used to detect BMD in a clinical situation, including quantitative ultrasound (QUS), dual-energy X-ray absorptiometry (DEXA), quantitative computed tomography (QCT), and dual-photon absorptiometry, but there are no reports definitively discussing which technique is better than the others.

Schizophrenia is one of the most severe psychiatric disorders in the world, and can lead to a great many complications and disability. There have been a lot of comorbidities discovered in patients with schizophrenia. Fracture and osteoporosis are 2 of the most common comorbidities reported in recent decades and have attracted much clinical attention.^2–4^ A recent one meta-analysis has proven that the schizophrenic patients have been at increased risk for fracture.^5^ Besides, the usage of antipsychotics would increase the risk of falls and fracture in numerous report. For example, the atypical antipsychotics seem to result in higher risk of fracture than traditional antipsychotics in report by Kolanowski et al.^6^ At the present time, there are a number of clinical studies that have discussed osteoporosis risk and BMD in patients with schizophrenia. Some of them suggest that the incidence of BMD in schizophrenia patients is significantly lower than that in healthy subjects.^7–11^ Among those studies supporting decreased BMD in patients with schizophrenia, some have done more specific investigation into sex differences. In those studies, both female and male schizophrenic patients had lower BMD than the healthy controls.^11–14^ Moreover, the researchers also tried to evaluate the effect of age,^9,11^ duration of illness,^7^ genetic risk,^10^ and exercise^14^ on BMD. At the same time, there have been some controversial reports published. Some have revealed no significant difference between BMD in patients with schizophrenia and in healthy controls,^15^ or no significant difference in pretreatment but significant difference after conventional antipsychotics treatment.^16^ Other reports suggest that BMD is significantly lower in patients with schizophrenia than in healthy controls, but only in females^17^ or that the changes in BMD have a sex-specific difference.^18^

In addition, Bushe et al^19^ have suggested that hyperprolactinemia caused by different antipsychotics would increase the risk of osteoporosis in schizophrenic patients, and that this might complicate research on the changes in BMD in schizophrenia. Most studies currently discussing this topic have divided their subjects according to the effect of prescribed antipsychotics on prolactin levels, that is, into groups of prolactin-raising (PR) antipsychotics or prolactin-sparing (PS) antipsychotics. Some of these studies have found significantly different BMD levels,^20–22^ but others have not.^23–26^ Therefore, there are no conclusive results as to whether the use of different antipsychotics would result in different changes in BMD in schizophrenic patients.

Despite the number of reports and articles, there is still limited evidence as to whether BMD in schizophrenia patients is lower than in healthy subjects. Oderda et al used a meta-analysis to investigate the risk of hip fracture related to psychotropics usage, but still could not reach a conclusion.^27^ Crews et al conducted another meta-analysis to clarify the divergent findings regarding BMD in patients with schizophrenia, but the main focus of their report was on the effect of antipsychotics treatment on BMD.^28^ Stubbs et al recently published a meta-analysis specifically discussing the changes in BMD in patients with schizophrenia.^29^ However, in that report, the authors mainly focused on the high prevalence of osteoporosis in schizophrenia and only briefly mentioned a comparison of BMD in schizophrenia patients and age- and sex-matched healthy controls (HCs). Besides, there was little discussion and investigation into the association of BMD and clinical variables in that report. In addition, the authors discuss little about the possible effect of age, one of the most important clinical moderators on the BMD, on the BMD in schizophrenia. In previous reports, BMD was believed to have gradually decreased significantly in different sites with cut-point of age 40 in both males and females.^30,31^ Furthermore, that study did not carry out a further detailed investigation into the possible effect of different antipsychotics on BMD, and the divergent effect on prolactin levels.

The aim of our study was to conduct a meta-analysis using a thorough and broad database search to investigate the changes in BMD in patients with schizophrenia and, at the same time, the possible risk factors or moderators affecting BMD in such patients, for example, age, sex, duration of treatment, and other variables. Furthermore, to thoroughly investigate the possible effects on BMD of different antipsychotics with divergent effects on prolactin levels, we conducted a meta-analysis of the changes in BMD in schizophrenic patients receiving different antipsychotics. On the other hand, we will focus more specifically on subjects with young age because there will be more economical loss when fracture happens in young age than in old age.

## METHODS

**FIGURE 1:**
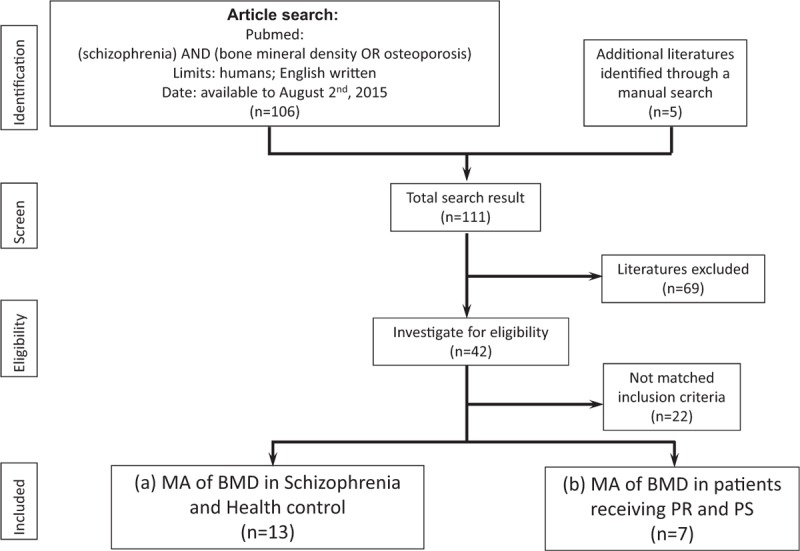
Flowchart of selection strategy of the current meta-analysis. BMD = bone mineral density; MA = meta-analysis; PR = prolactin-raising antipsychotics; PS = prolactin-sparing antipsychotics.

We conducted a computerized search on the PubMed database using the keywords (schizophrenia) AND (bone mineral density OR osteoporosis) with the limitation of “human study” and “English written.” To avoid possible bias during the search and selection of eligible articles, the search process was conducting by 3 psychiatrists, Tseng PT, Cheng YS, and Tu KY. The search period was from the date available online to August 2, 2015. The search strategy is depicted in Figure 1. Initially, we excluded articles without a relationship to osteoporosis or BMD in schizophrenia. The inclusion criteria were as follows: articles with relevance to comparisons of BMD in patients with schizophrenia and healthy controls, or articles discussing comparisons of BMD in patients receiving PS and PR; articles about clinical trials; therefore, we excluded all articles without comparison to HCs or articles without comparison of antipsychotics. Review articles and case reports were excluded. Those articles with commentary contents were also excluded. Finally, we divided the remaining articles into 2 categories: articles discussing the difference in BMD in schizophrenic patients and HCs and those discussing the difference in BMD in schizophrenic patients receiving PR or PS. The results of the literature search and the reports that are included in our study are listed in Table 1 a for (a) and Table 1 b for (b).

**TABLE 1:**
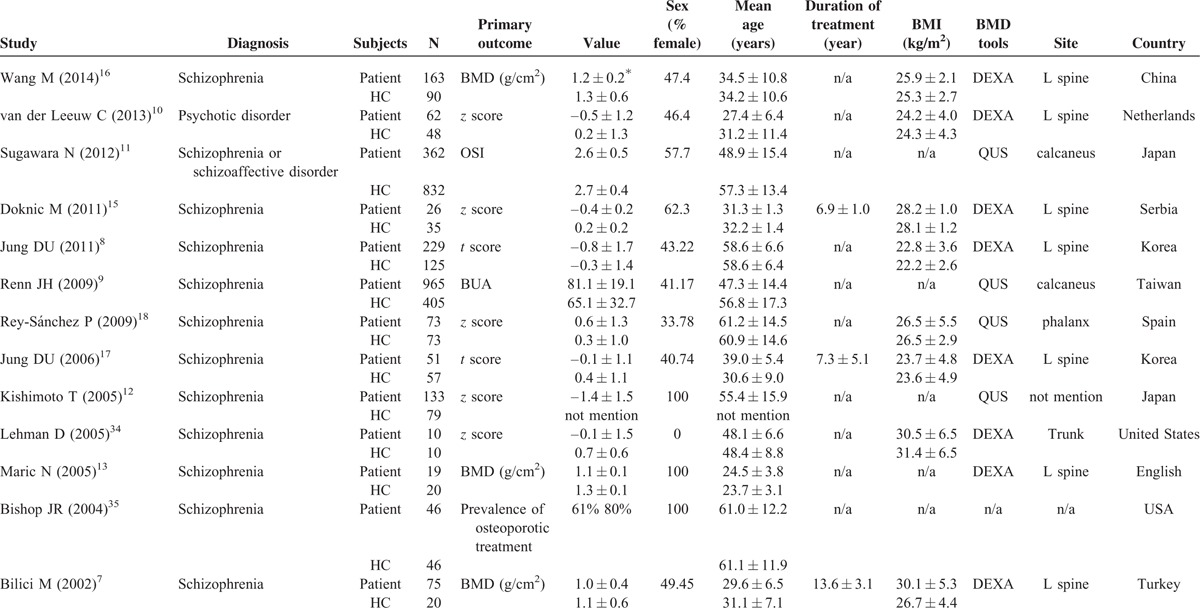
Summary of Characteristics of Studies in Current Meta-Analysis of Comparison of BMD (A) Between Schizophrenic Patients or Healthy Controls and (B) in Schizophrenic Patients Receiving PR and PS

**TABLE 1 (Continued):**
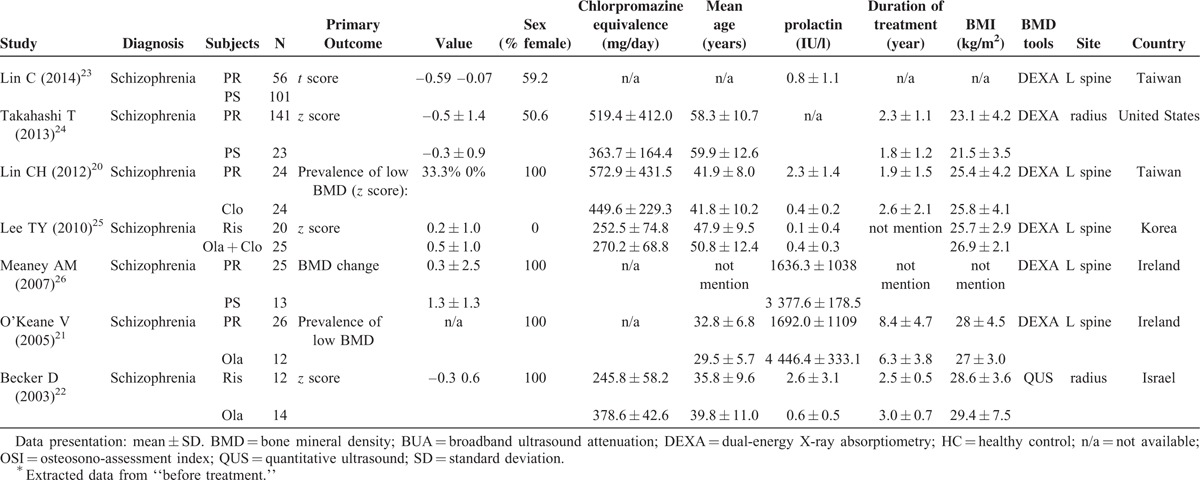
Summary of Characteristics of Studies in Current Meta-Analysis of Comparison of BMD (A) Between Schizophrenic Patients or Healthy Controls and (B) in Schizophrenic Patients Receiving PR and PS

In this study, we set the primary outcome as BMD, checked by DEXA, QUS, QCT, or dual-photon absorptiometry. We extracted the data on BMD from the remaining articles in the order of *z* score, *t* score, and finally, the absolute data. Since the *z* score is based on an age- and sex-matched population and the *t* score is based on a young-adult population only, we considered the *z* score to be clinically more relevant than the *t* score and absolute data of BMD. In the recruited articles, the BMD studies were conducted at many different sites, such as the lumbar spine (L-spine), femur, and the digits. We found that the BMD examinations in most articles were conducted at the L-spine, and then the femoral necks or trochanters. Therefore, we extracted all BMD data for the L-spine first, followed by the femoral neck or trochanter, for 2 reasons: the osteoporotic fractures occurred mostly in the vertebrae^32^ so the BMD of the vertebrae would be most relevant to clinical settings, and most studies have used this site for the BMD examination. Furthermore, since there was a lack of evidence as to whether the DEXA is more sensitive and specific than the QUS, QCT, or dual photon absorptiometry, we extracted the BMD data yielded by the most commonly used technique. The DEXA, followed by QUS, was the most frequently used in all the articles to detect BMD; so, we extracted the BMD data of the DEXA first, and then that of the QUS. In order to clarify any possible bias in terms of techniques selected in different studies, we subdivided the studies and performed another meta-analysis based on the tools used in the studies.

In addition to the comparison of BMD in patients with schizophrenia and HCs, we also tried to investigate the difference in BMD of schizophrenic patients receiving PR and PS. The classification of PR and PS is made according to the previous reports.^23–25^ Therefore, the PR is defined as antipsychotics with prominent prolactin-raising effect, including first-generation antipsychotics, risperidone, paliperidone, amisulpride, or ziprasidone; the PS is defined as antipsychotics without or with minimal prolactin-raising effect, including clozapine, olanzapine, quetiapine, or aripiprazole.

We calculated the effect sizes (ESs) of the individual studies recruited for our meta-analysis through standardized mean differences with Hedges's adjusted *g*. We calculated all the results of the studies using the random-effects model. The confidence interval was defined as 95%. The significance of the pooled ES was determined by the *z* test. We used Q statistics to examine the homogeneity of the ES distribution. If the result of the Q statistic was rejected, this would suggest that the ES distribution might be heterogeneous. We used Egger's regression to examine for possible publication bias. Furthermore, to investigate the possible confounding effect by the clinical variables, we perform the procedure of meta-regression with the fixed effect regression. We tried to contact the authors as possible if the detailed data are unavailable through the literatures.

We performed the meta-analysis using Comprehensive Meta-Analysis, Version 2 (Biostat, Englewood, NJ) software. Two-sided significance was set as a *P* value <0.05. The current meta-analytic procedure fulfilled with the criteria of Preferred Reporting Items for Systematic reviews and Meta-Analyses (PRISMA) compliant.^33^ In addition, in current meta-analysis, the ethical approval was not necessary because we did not deal with the actual patients’ personal data and there were no patients being harmed due to our procedure of meta-analysis.

## RESULTS

Using our search strategy, we initially included 111 articles, of which 69 were excluded because of their irrelevance to osteoporosis or BMD in schizophrenia. We screened the remaining 42 articles using the inclusion and exclusion criteria introduced in the Methods section. A total of 13 articles were finally included in the meta-analysis for comparison of BMD in patients with schizophrenia and HCs, and 7 articles were included for comparison of BMD in schizophrenic patients receiving PR and PS. The results of the literature selection included in our study are listed in Table 1 a for (a) and Table 1 b for (b).^7–13,15–18,20–26,34,35^

### BMD in Patients With Schizophrenia and Healthy Controls

**FIGURE 2:**
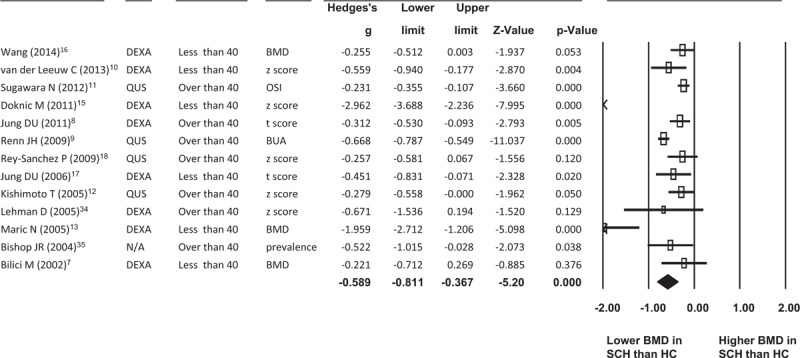
Forest plot of the MA for comparison of the BMD in SCH and HCs. BMD = bone mineral density; BUA = broadband ultrasound attenuation; DEXA = dual-energy X-ray absorptiometry; HC = healthy controls; MA = meta-analysis; N/A = not applicable; OSI = osteosono-assessment index; QUS = quantitative ultrasound; SCH = schizophrenia.

Eight reports were recruited in that described the numbers of subjects;^7–9,13,15,17,18,34^ in all, a total of 2214 patients with schizophrenia (mean age (mean ± standard deviation (SD)) = 47.3 ± 15.5) and 1840 HCs (mean age (mean ± SD = 53.5 ± 16.5) were recruited. Two studies in the meta-analysis of comparison of BMD in patients with schizophrenia and HCs did not show the numbers of subjects.^12,35^ The meta-analysis for comparison of BMD in patients with schizophrenia and HCs revealed significantly lower BMD in patients with schizophrenia than in the HCs (ESs = –0.589, 95% confidence interval (95% CI): –0.811∼ –0.367, *P* < 0.001) (Fig. 2). Besides, using Egger's regression analysis (*P* = 0.129 in 1-tailed and *P* = 0.258 in 2-tailed), we could not find a significant publication bias.

### Meta-Analysis of Studies Using Different Tools to Investigate BMD

**FIGURE 3:**
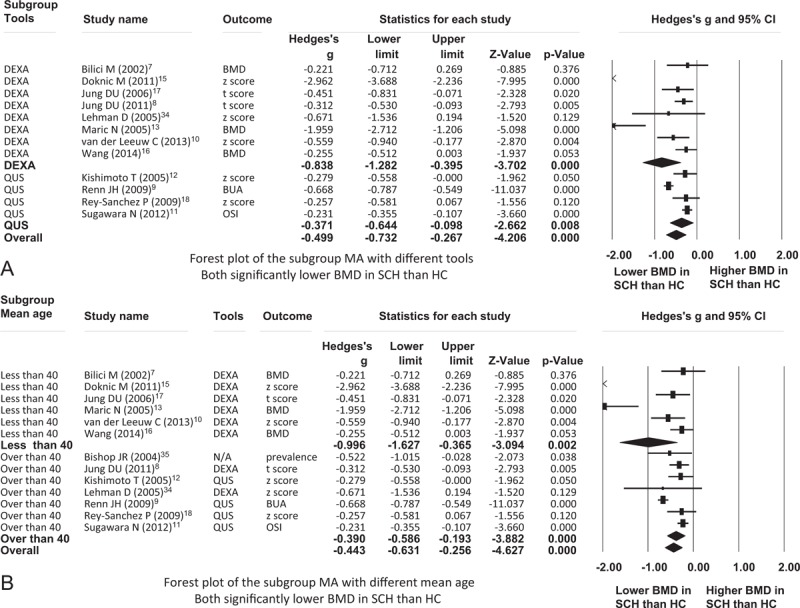
(A) Forest plot of the subgroup MA with different tools. (B) Forest plot of the subgroup MA with different mean age. BMD = bone mineral density; BUA = broadband ultrasound attenuation; DEXA = dual-energy X-ray absorptiometry; HC = healthy controls; MA = meta-analysis; N/A = not applicable; OSI = osteosono-assessment index; Psy = psychotic disorder; QUS = quantitative ultrasound; SCH = schizophrenia.

To clarify any possible differences in results between BMD investigated using different tools, we performed further subgroup meta-analysis of the studies carried out with different tools individually. One report did not identify the tool used to investigate BMD, in which study the authors screened the osteoporosis by reviewing the electronic records.^35^ In all 8 studies using DEXA, we found a similar result: BMD in patients with schizophrenia was significantly lower than in HCs (ESs = –0.838, 95% CI = –1.282∼ –0.395, *P* < 0.001). On the other hand, in 4 studies using QUS, we found similar significant lower BMD in patients with schizophrenia than in HCs (ESs = –0.371, 95% CI = –0.644∼ –0.098, *P* = 0.008) (Fig. 3A).

### Meta-Regression for Clinical Variables

At the same time, we investigated the possible moderators for BMD in this situation using meta-regression. We found that sex does not have a significant effect on BMD (point estimate of slope: −0.0004, standard error: 0.003, *P* value = 0.90), but age and duration of treatment could have a significant moderating effect on BMD (point estimate of slope: 0.014, standard error: 0.004, *P* value < 0.001 in former; point estimate of slope: 0.13, standard error: 0.047, *P* value = 0.0051 in later). The *P* value of either the Q (model) or the Q (total) of meta-regression of the age and duration of treatment effect on BMD achieved significance (*P* < 0.001, *P* < 0.001 separately in former; *P* < 0.001, *P* < 0.001 separately in later), which indicates the heterogeneity among studies included in this meta-regression. On the other hand, we could not perform analytic procedure of meta-regression for some other variables, including body mass index (BMI), body weight, duration of disease, serum prolactin levels, and % patients with hyperprolactinemia, because of lack of detailed data.

### Meta-Analysis in Different Age Subgroups

The BMD was believed to have gradually decreased significantly alone with age with cut-point of age 40 in both males and females.^30,31^ Therefore, we subdivided those studies into those with mean age below or above age 40 and performed meta-analysis of these 2 age-specific subgroups. In the mean age below 40 subgroup, BMD of patients with schizophrenia was still strongly and significantly lower than that of HCs (ESs = –0.996, 95% CI = –1.627∼ –0.365, *P* = 0.002). In the mean age above 40 subgroup, BMD was significantly different between patients with schizophrenia and HCs (ESs = –0.390, 95% CI = –0.586∼ –0.193, *P* < 0.001) (Fig. 3B).

We tried to perform the procedure of meta-regression with the item of “mean age,” “female sex proportion,” and “duration of treatment” in the subgroup meta-analysis of different age. The result of meta-regression revealed significant association between the mean age, female sex proportion, and duration of treatment and the BMD in the younger age subgroup (point estimate of slope: 0.06, standard error: 0.02, *P* value = 0.004; point estimate of slope: −0.04, standard error: 0.007, *P* value < 0.001; point estimate of slope: 0.13, standard error: 0.047, *P* value = 0.005, separately); on the other hand, the result of meta-regression showed significant association only between the mean age, and female sex proportion and the BMD in the older age subgroup (point estimate of slope: 0.04, standard error: 0.01, *P* value < 0.001; point estimate of slope: 0.008, standard error: 0.003, *P* value = 0.01, separately). The procedure of meta-regression for duration of treatment could not perform in older age subgroup because of lack of detailed data.

Among these 2 subgroup analyses, we found out heterogeneity of the data source in older age subgroup. In this subgroup, the BMD was obtained through variable tools, including DEXA^34^ and QUS^11,12^ at site of L-spine,^8^ calcaneus,^9^ and digits.^18^ On the other hand, the data sources are more uniform in the younger age subgroup; all the data of BMD were obtained through DEXA at site of L-spine.^7,10,13,15–17^

### Meta-Analysis for PR and PS

**FIGURE 4:**
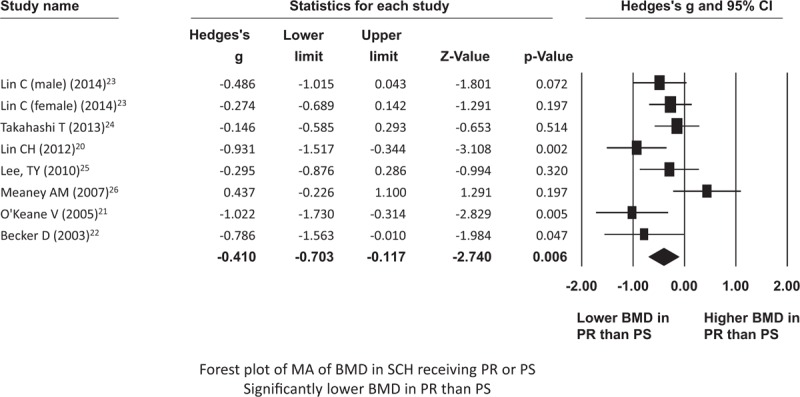
Forest plot of MA of BMD in SCH receiving PR or PS. BMD = bone mineral density; PR = prolactin-raising antipsychotics; PS = prolactin-sparing antipsychotics; SCH = schizophrenia.

In all, a total of 304 schizophrenic patients receiving PR (mean age (mean ± SD) = 51.4 ± 13.9) and 212 schizophrenic patients receiving PS (mean age (mean ± SD) = 46.6 ± 14.7) were recruited. In the meta-analysis of comparison of BMD in schizophrenic patients receiving PR and PS, we found that the BMD in schizophrenic patients receiving PR is significantly lower than that in schizophrenic patients receiving PS (ESs = –0.410, 95% CI = –0.703∼–0.117, *P* = 0.006) (Fig. 4). In addition, the funnel plot and Egger's test revealed that there was no significant publication bias (*P* = 0.39). In this part of the meta-analysis, we performed meta-regression with female proportion, mean age, BMI, and duration of treatment. There was a significant association between BMD and mean age (slope = 0.036, *z* value = 2.61, *P* value = 0.009) and between BMD and BMI (slope = –0.12, *z* value = –1.99, *P* value = 0.046). However, there was no significant association between BMD and female proportion/duration of treatment (data not shown). On the other hand, in order to clarify the possible confound effect of the antipsychotics dosage, expressing as the chlorpromazine equivalence, and the serum prolactin levels on the BMD, we also tried to investigate it through meta-regression. In the meta-regression of chlorpromazine equivalence and serum prolactin levels, we could not find out any significant association between them and the BMD (*P* = 0.916 and 0.456, separately).

## DISCUSSION

To our knowledge, this is the first meta-analysis to investigate the difference in BMD in schizophrenia patients and HCs in different age subgroups and the effect of antipsychotics use and other possible moderators of BMD. In addition, not only focusing on the possible moderating clinical factors, we also investigate the possible confounding effect by the different tools and sites of BMD examination. The main findings in our meta-analysis are there was significantly lower BMD in patients with schizophrenia than in HCs, the differences between schizophrenic patients and HCs were both significant in the 2 age-subgroups or 2 different detection tools, and BMD was significantly lower in schizophrenic patients receiving PR than in those receiving PS.

Results in the recent meta-analysis conducted by Stubbs et al revealed that BMD was significantly lower in patients with schizophrenia than in HCs and this was associated with male sex and age.^29^ The results of our study are generally in agreement with the above. However, in our study, we further investigated the effect of age and antipsychotics, especially PR and PS, on BMD in patients with schizophrenia and HCs. We subdivided studies according to their mean age; we found that the differences between schizophrenic patients and HCs were still significant after subdividing into the 2 age-subgroups. We also found that BMD in the schizophrenic patients receiving PR was significantly lower than in those receiving PS.

In this study, meta-analysis revealed that BMD was significantly lower in schizophrenic patients than in HCs. Some reports have discussed the possible risk factors for or etiology of low BMD or osteoporosis in patients with schizophrenia.^2,3^ There are many possible reasons or causes that may offer an explanation for our results. Medication, especially antipsychotics, might be one of the possible causes. In fact, in our meta-analysis, BMD in schizophrenic patients receiving PR was significantly lower than that in patients receiving PS. The *z* scores of BMD in the patients receiving either PR or PS were generally lower than in age- and sex-specific normal populations; that is, both *z*-scores were lower than zero. There are some reports suggesting that hyperprolactinemia induced by prescribed antipsychotics or by any other factor would have an impact on BMD in such patients;^4,36^ this hypothesis can be at least partially explained by the result of our meta-analysis of comparison of BMD in schizophrenic patients receiving PR and PS, in which BMD was significantly lower in schizophrenic patients receiving PR than in those receiving PS. However, there are some controversial findings regarding the negative effect of prolactin on BMD in schizophrenic patients.^37,38^ Meaney et al found a similar effect, in which long-term usage of PR was associated with decreased BMD.^39^ Another possible explanation of this phenomenon is oxidative stress. More and more evidence suggests that schizophrenia is caused not only by genetics but also by oxidative stress. In the glutathione study, researchers found a significant glutathione decrease in the cerebrospinal fluid of schizophrenic patients, compared with HCs.^40^ Others have found that the glutathione deficit might be implicated in early-onset first episode psychosis.^41^ Oxidative stress has been proved, at least partially, to have an impact on osteoporosis in human.^42^ However, add-on anti-oxidants would prevent the risk of osteoporosis or bone loss in human subjects.^43^ In addition, the negative symptoms of schizophrenia would result in the loosening lifestyle, a poor nutrition status, and the abolition of exercising, which are believed to be risk factors for osteoporosis or low BMD. Similarly, evidences have proven that schizophrenic patients have generally poor diet and numerous metabolic problems, which all result in risk of osteoporosis.^44^ Besides, the inadequate calcium intake, reduced levels of vitamin D, and high levels of smoking would contribute to the low BMD in schizophrenic patients.^29^ Some evidence has shown the benefit of exercise on bone health.^45^ However, at the present time, there is still a lack of definite evidence to prove the direct or indirect relationship between the possible causes mentioned above and the low BMD or osteoporosis in schizophrenic patients. Therefore, in the future, we need more studies to prove the possible pathophysiology or mechanism of low BMD in young schizophrenic patients who are drug-naive or who only received short-term psychotropic agents.

Our study implicates the clinical importance of the significantly lower BMD in schizophrenia patients than in HCs. In fact, lower BMD or osteoporosis is believed to increase the risk of fractures.^46^ In another meta-analysis conducted by Oderda et al, the risk of hip fractures in an older adult population also increased with association with usage of first- and second-generation antipsychotics.^27^ Therefore, patients with schizophrenia actually have a high risk of fracture. At the present time, more and more methods are being introduced to improve BMD or osteoporosis in patients with or without hip fracture, including denosumab, vitamin D supplements, and other types of nonpharmacological management.^47,48^ However, there is still a lack of evidence to prove the effectiveness and safety of such management in schizophrenic patients.

Our main finding in our study highlights another important point that both the different levels of BMD in these 2 age-subgroups are significant. Here comes 1 problem that in these 2 age-subgroups the heterogeneity varies. In the older age-subgroup, just as mentioned in the section of Result, the BMD was obtained through variable tools, including DEXA and QUS at site of L-spine, calcaneus, and digits. Therefore, the result of meta-analysis would be complicated by such many confounding factors. On the other hand, the data sources are more uniform in the younger age subgroup, which all were obtained through DEXA at the site of L-spine. Besides, there would be more economical loss when the hip or spine fracture happened in younger subjects than in older subjects. So, we focused on the result of meta-analysis in younger age-subgroup not only for economic reason but also for the reason of less confounding factors. In this part of meta-analysis, we found out significant positive association with mean age, and duration of treatment with BMD but inverse association with female sex proportion. This phenomenon would conflict with the previous evidence of decreasing BMD alone with aging.^9,49^ Interestingly, this phenomenon could be supported by another 1 report conducted in a huge community study in Taiwan. Renn et al had indicated the different extent of changes in BMD with age in schizophrenia patients and HCs. A significantly negative trend of mean BMD with age was found in community controls, but not in schizophrenic patients. In addition, in the picture depicted by Renn, the BUA would increase alone with aging in schizophrenic patients although not always significantly.^9^ However, in the current reports, we still lack definite evidence to explain this phenomenon. In addition, there is 1 possible confounding factor that is the duration of antipsychotics treatment. As our part of result of meta-analysis, the usage of antipsychotics would affect the BMD in schizophrenic patients. In current evidence, we found out significant association between the BMD and the duration of treatment.

Here comes another question that, in current meta-analysis, we chose the DEXA as the first choice of BMD detection. However, it might be questionable if the different tools applied in detection of BMD would have possible confounding factor or not. In previous studies, some authors tried to investigate the implacability of the DEXA, QUS, and other tools in the prediction of osteoporosis or the risk of fracture. However, there was no consensus regarding which one is better than the others. Evidences have reported that the DEXA and QUS have significantly different sensitivities and specificities.^50,51^ Because of a number of advantages of the DEXA, the DEXA of the spine currently is considered a “gold standard” diagnostic tool for BMD examination.^52^ Therefore, for clinicians, it is important to be careful of the possible risk of bias when reading articles using QUS as the tool for examining BMD. In this study, we tried to evaluate the possible bias with different tools. We subdivided those studies into those with DEXA and those with QUS. We found that BMD was still significantly lower in patients with schizophrenia than in HCs that were examined by DEXA. On the other hand, in the subgroup meta-analysis of studies with QUS, although the pooled ESs revealed significantly lower BMD in schizophrenic patients than in healthy controls, this evidence was limited because of the only 4 studies included this subgroup meta-analysis. Furthermore, when we investigate the results in these 4 studies conducted with QUS, we found that there was similarly lower BMD in both female schizophrenic patients and HCs in the report by Kishimoto,^12^ and lower BMD in schizophrenic patients of both sexes than that in HCs in the study conducted by Sugawara.^11^ Similar results could not be found in the other 2 reports. In the report by Rey-Sánchez et al, the changes in BMD in schizophrenic patients were the opposite of that of the report by Kishimoto and Sugawara^18^ and in the report by Renn et al, the difference in BMD between schizophrenic patients and HCs would vary according to age and sex.^9^ Therefore, we still need to be careful when interpreting such data, especially those studies that used the QUS. It is important to be careful of the possible confounding factors mentioned above when applying our results to clinical practice.

Finally, we would like to indicate a new potential direction for studies in the future. At present, most studies on BMD in schizophrenic patients have focused on BMD of the L-spine, trochanter, phalanx, radius, or calcaneus;^7,9,11–13,16,18,24,38,53,54^ only a few have investigated BMD in schizophrenic patients at the proximal femur or hip.^8,10,26,37^ As mentioned above, there is a consensus that hip and spine DEXA a “gold standard” diagnostic tool for osteoporosis.^52^ Although there has been no conclusive report discussing the most frequent fracture site in schizophrenic patients, the most widely studied fracture site in these patients nowadays is the hip.^2,55^ Hip fracture is also considered to have a high correlation with hyperprolactinemia and antipsychotics usage in such patients.^2,19^ In fact, there is evidence that DEXA at the lumbar spine and proximal femur is most correlated with spinal and hip fractures.^3^ We suggest that researchers pay more attention to and focus on BMD investigation by DEXA, taken at the site of the proximal femur, which would be more relevant and close to clinical practice.

## LIMITATION

There are some limitations that should be mentioned before applying our results to clinical practice. The first is that we subdivided the studies according to the overall mean age of the studies rather than according to the selection criteria in every study; this might have led to some bias in the meta-analysis result. Besides, our study can only point to the phenomenon that BMD in schizophrenic patients is lower than that in healthy controls, and cannot reveal any possible pathophysiology or mechanism of this phenomenon. In addition, in the meta-analysis of comparison of BMD in patients with schizophrenia and HCs, we could not rule out the possible effect of medication on BMD, since it seems to have had an impact on BMD in the schizophrenic group, based on the results of the meta-analysis of comparison of BMD in schizophrenic patients receiving PR and PS. Although we tried to investigate the possible effect of antipsychotics on BMD, we still could not completely distinguish the effect on BMD of schizophrenia itself or antipsychotics use. Studies have tried to investigate the changes in BMD in schizophrenic patients who are drug-free or only receiving a short course of antipsychotics. However, the findings were controversial: one revealed significantly lower BMD in psychotic patients^13^ and another revealed insignificant changes.^16^ Besides, in the meta-analysis of comparison of BMD in schizophrenic patients receiving PR and PS, we try to compare the difference in BMD in schizophrenic patients receiving different categories of antipsychotics through dividing them as those receiving PR or PS. However, there is 1 problem that, actually, all the antipsychotics would result in the hyperprolactinemia, in extent of more or less.^56^ In addition, the dosage of each antipsychotics would alter the prolactin levels, too. However, in current meta-analysis, there is no significant association between these 2 confounding factors and the BMD in schizophrenic patients. This might be, at least partially, resulted from the small sample size. Last but not least, in the section of meta-analysis in different age subgroups, we could find significant association between duration of treatment and the BMD in younger age subgroup, but we could not perform similar investigation in the older age one because of lack of related data. This might implicate confounding effects on the result of meta-analysis.

## CONCLUSION

Despite the above limitations, our study still has some important implications for clinical practice. This report reminds us of the importance of possible comorbidity of schizophrenia, especially osteoporosis, when treating these patients. This is especially important when dealing with those receiving prolactin-raising antipsychotics.
